# *Stepping Stones or Second Class Donors?:* a qualitative analysis of gay, bisexual, and queer men’s perspectives on plasma donation policy in Canada

**DOI:** 10.1186/s12889-021-10480-x

**Published:** 2021-03-05

**Authors:** Daniel Grace, Mark Gaspar, Benjamin Klassen, David Lessard, Praney Anand, David J. Brennan, Nathan Lachowsky, Barry D. Adam, Joseph Cox, Gilles Lambert, Jody Jollimore, Trevor A. Hart

**Affiliations:** 1 Dalla Lana School of Public Health, University of Toronto, 155 College Street, Toronto, ON M5T 3M7 Canada; 2 Community-Based Research Centre, 1007-808 Nelson Street, Vancouver, BC V6Z 2H2 Canada; 3 Centre for Health Outcomes Research, McGill University Health Centre, 5252 de Maisonneuve West, Montréal, QC H4A 3S5 Canada; 4 Factor-Inwentash Faculty of Social Work, University of Toronto, 246 Bloor St W, Toronto, ON M5S 1V4 Canada; 5 School of Public Health & Social Policy, Faculty of Human & Social Development, University of Victoria, P.O. Box 1700, STN CSC, Victoria, BC V8W 2Y2 Canada; 6 Department of Sociology, Anthropology, and Criminology, University of Windsor, 401 Sunset Ave, Windsor, ON N9B 3P4 Canada; 7 Direction de santé publique de Montréal, CIUSSS du Centre-Sud-de-l’Île-de-Montréal, 1560 Sherbrooke St E, Montreal, QC H2L 4M1 Canada; 8 Department of Psychology, HIV Prevention Lab, Ryerson University, 350 Victoria St, Toronto, ON M5B 2K3 Canada

**Keywords:** Gay, bisexual, queer and other men who have sex with men, Blood donation, Plasma donation, Policy, Qualitative, Discrimination, HIV/AIDS, Canada

## Abstract

**Background:**

Men who have sex with men (MSM) are not eligible to donate blood or plasma in Canada if they have had sex with another man in the last 3 months. This time-based deferment has reduced since 2013; from an initial lifetime ban, to five-years, one-year, and now three-months. Our previous research revealed that gay, bisexual, queer, and other MSM (GBM) supported making blood donation policies gender-neutral and behaviour-based. In this analysis, we explored the willingness of Canadian GBM to donate plasma, even if they were not eligible to donate blood.

**Methods:**

We conducted in-depth interviews with 39 HIV-negative GBM in Vancouver (*n* = 15), Toronto (*n* = 13), and Montreal (*n* = 11), recruited from a large respondent-driven sampling study called Engage. Men received some basic information on plasma donation prior to answering questions. Transcripts were coded in NVivo following inductive thematic analysis.

**Results:**

Many GBM expressed a general willingness to donate plasma if they became eligible; like with whole blood donation, GBM conveyed a strong desire to help others in need. However, this willingness was complicated by the fact that most participants had limited knowledge of plasma donation and were unsure of its medical importance. Participants’ perspectives on a policy that enabled MSM to donate plasma varied, with some viewing this change as a “stepping stone” to a reformed blood donation policy and others regarding it as insufficient and constructing GBM as “second-class” donors. When discussing plasma, many men reflected on the legacy of blood donor policy-related discrimination. Our data reveal a significant *plasma policy disjuncture—*a gulf between the *critical importance* of plasma donation from the perspective of Canada’s blood operators and patients and the feelings of many GBM who understood this form of donation as less important.

**Conclusions:**

Plasma donor policies must be considered in relation to MSM blood donation policies to understand how donor eligibility practices are made meaningful by GBM in the context of historical disenfranchisement. Successful establishment of a MSM plasma donor policy will require extensive education, explicit communication of how this new policy contributes to continued/stepwise reform of blood donor policies, and considerable reconciliation with diverse GBM communities.

## Background

While motivations, deterrents, and the social meanings attributed to whole blood donation have received considerable scholarly attention [1–9] much less is known about the donation of plasma, particularly when non-remunerated [10–14]. The transfusion of plasma—the liquid component of blood, excluding red and white blood cells, and platelets—is used for individuals with chronic and genetic conditions such as bleeding disorders, burns, and immunodeficiency. The protein-rich component of plasma is essential for manufacturing treatment drugs and therapies [15, 16].

The medical importance of plasma in making a lifesaving difference for patients is part of Canada’s blood operators’ messaging to potential donors [15, 16]. Canadian Blood Services has also expressed its commitment to maintaining the safety of the supply while increasing national plasma sufficiency, arguing that there is a “looming threat to the supply of plasma” within a national and international context where “demand for plasma is rapidly growing” [17].

Canadian Blood Services and its sister blood operator organization in the province of Québec, Héma-Québec, do not compensate donors for any form of blood donation, including plasma donation.^1^ Plasma is collected in two different ways. In what is called “recovered plasma”, plasma and other blood components are separated from whole blood post-donation. This plasma collected from “recovered” plasma is used for both transfusion and fractionation. In “source plasma” (or “plasmapheresis” donation), an apheresis machine extracts plasma from whole blood and returns components such as platelets and red and white blood cells back into the donor.^2^ While it is a much less common form of donation in Canada at present, since a higher volume of plasma can be collected each time, plasmapheresis (source) donation is considered more efficient for the blood system. All plasma collected is blood-typed and tested for pathogens such as HIV and the hepatitis C virus; the plasma is subsequently frozen for distribution to hospitals [15, 22].

Within the published plasma donation research, little is known about the willingness of men who have sex with men (MSM)^3^ to donate plasma if eligible. Research with potential MSM donors has, like research conducted with the general population, focused on whole blood donation, including the considerable policy debates and activism surrounding the deferment of MSM from blood donation in many countries and alternative screening procedures [5, 6]. A notable exception is the recent work of Caruso et al. [23] who conducted qualitative research in Montreal, Canada, to examine the operational feasibility and overall acceptability of an MSM program for plasma donation through Héma-Québec. While focus group participants expressed some interest in becoming plasma donors if eligible, some “were left bitter and afraid that the [plasma] programme would be used to sidestep MSM’s demands regarding whole blood donation, and to end discussions with LGBTQ+ [lesbian, gay, bisexual, trans, queer] communities” [23].

In Israel, Levy et al. [24] found high acceptability among MSM for participation in the Frozen Plasma Quarantine Policy (FPQP) which would give MSM the opportunity to donate plasma that would be frozen, quarantined, and released after 4 months only if a subsequent donation was negative for HIV and other sexually transmitted infections (STIs). 64.5% of participants said they would like to donate through this policy as opposed to only 10.4% who indicated a preference for a 12-month deferral policy. Levy et al. note that the goal of this plasma policy option was “to accede to the wishes of the LGBT community members by welcoming them as blood donors, without compromising blood safety” [24]. This survey was completed between June–July 2017 after Israel eliminated the lifetime ban on MSM donating blood earlier that same year.

Currently, men in Canada cannot donate blood or plasma if they have been sexually active (oral or anal intercourse) with another man in the last 3 months [15]. This sexual behaviour time-based deferment policy has decreased over time, from a lifetime ban introduced in 1983 in response to the HIV/AIDS epidemic, to five-years (2013), one-year (2016), and now to a three-month deferment (2019) [5, 25, 26]. These trends in Canada are in keeping with recent policy changes in France, Japan, Denmark, the United States, and the United Kingdom, that have shifted from indefinite bans to time-based deferments of between 3 to 6 months [6, 27].

Our previous analysis of the views of gay, bisexual, queer, and other men who have sex with men (GBM) [6] concerning blood donation policy and willingness to donate blood if eligible has drawn on theories of *biological citizenship* [28, 29] and *sexual citizenship* [30, 31]. We found complex connections across these forms of citizenship for our participants. Most relevant to our present analysis, we observed that the majority of men we interviewed understood themselves to be “safe” or “low risk” potential blood donors who were interested in blood donation. Our participants considered blood donation to be associated with an important form of altruistic civic engagement that would allow them to help their communities and also feel good about being a donor [6]. The men we interviewed “considered blood donation deferrals for MSM an affront to issues of equity and an expression of systemic homophobia” [6]. Furthermore, our study participants overwhelmingly supported making blood donation policies gender-neutral and risk behaviour-based as opposed to blanket MSM-specific time-based deferments [5].

Building on our previous analysis and amid a policy and research environment focused largely on blood donation deferral for MSM, we examined the willingness of GBM to donate plasma, even if they were not able to donate whole blood. As none of our participants were active blood donors at the time of the research, our analysis is different from studies which have sought to understand how to “convert” current blood donors into plasma donors [11, 14]. Our objective was to inductively explore if a demographically diverse sample of GBM living in Vancouver, Toronto, and Montreal would be willing to donate plasma if they were eligible. We focused our analysis on attitudes related to changes in plasma donor eligibility given that at the same time Canadian Blood Services was in the process of assembling a proposal for Health Canada related to plasma donation. As Vesnaver et al. [32] put it, these policy activities represent an “effort to be more inclusive while maintaining the safety of the blood supply, including some [GBM] that are at low to no risk of HIV infection such as those in monogamous relationships.”

## Methods

### Recruitment

Between March–October 2018, we recruited and conducted in-depth interviews with 39 HIV-negative GBM from a large respondent-driven sampling study called Engage. Engage is a longitudinal study of GBM living in Canada’s three largest cities: Vancouver, Toronto, and Montreal. This recruitment strategy allowed us to purposively invite a diverse sample of participants based on their responses to a comprehensive quantitative survey which included questions related to sociodemographic (particularly in relation to age, race, and gender identity) and behavioural factors associated with the occurrence of HIV.^4^ We prioritised recruiting GBM (61% of the sample) with a lower likelihood of HIV acquisition and thus those who would be more likely to be eligible to donate blood or plasma in the future [6]. We used quota sampling to ensure we spoke to GBM of different age groups across the three cities, and identified with a variety of ethno-racial backgrounds and gender identities, including transgender men [6].

The Engage study relied on the expertise of community engagement committees (CECs) composed of key stakeholders in GBM health, including service providers, advocates, and community organizers. Through quarterly meetings, CEC members provided input on our interview questions, recruitment strategy, analysis process, and knowledge translation. We received research ethics board approval from the University of Toronto, Ryerson University, the University of Windsor, McGill University, the University of British Columbia, Simon Fraser University, and the University of Victoria. Participants received a $30 CAD honorarium for taking part in these qualitative interviews.

Participants were invited by email to participate in interviews if they indicated a willingness to be contacted for future research after they had completed the quantitative and biomedical components of the Engage study. Across city sites, 97% of baseline study participants indicated that they were willing to be contacted.

### Interviews

Individual interviews were conducted in-person at university campuses, study offices, or community-based organizations in each city. In Vancouver and Toronto, interviews were conducted in English; in Montreal, interviews were conducted in English or French. Prior to beginning the interviews, participants provided written informed consent. Interviews were 30 to 90 min in duration and were audio recorded.

A primary focus of our interviews was understanding participants’ policy perspectives on blood donation for MSM [5], including their willingness to donate if eligible [6]. A secondary component of the interview, which is the focus of the present analysis, was exploring participants’ willingness to donate plasma if they were eligible. Participants were first asked how familiar they were with plasma donation. After a brief explanation of the plasma donation process, participants were asked: *How much would you say you desire to donate plasma in the future? How important is being able to donate plasma to you?*

After this, the interviewer explained a hypothetical policy for MSM to donate plasma even if their sexual practices prohibited them from donating blood:

*Because plasma donations can be stored for much longer than regular blood donations, it is possible to do additional testing on plasma at a later date to confirm the donation’s safety before it is supplied to those in need. Because of these extra safety mechanisms, one potential blood donation policy being explored is allowing HIV negative/status unknown gay men and other men who have sex with men to donate plasma even if they have had sex more recently and would not be able to donate blood. This process would involve having to donate plasma multiple times for eligibility. What are your thoughts on the ability to donate plasma even if you can’t donate whole blood? Under this policy shift, would you be interested in donating plasma?*

We have previously published a copy of the full interview guide [5].

### Analysis

Our analysis was informed by several key principles (or “decisions”) as outlined by Braun and Clarke’s review of thematic analysis [35]. The analysis was inductive, being directly linked to the data rather than being informed by a pre-existing theoretical framework. We focused on providing an in-depth account of one key aspect of the data set (i.e., plasma donation) rather than summarizing the data set in its entirety. The analysis involved identifying both semantic and latent (or interpretive) themes; however, our analytic focus here involved a close reading of the content of participant’s accounts to understand their motivations, opinions, and expressed meanings of plasma donation. Our related analysis of these interviews on *biological citizenship* and *sexual citizenship* used a constructive approach to investigate these underlying assumptions and to critically understand the politics of blood donation [6].

In concrete terms, interviews were transcribed verbatim, de-identified, and reviewed for accuracy. We used QSR NVivo software to assist in the analysis process of the transcripts using thematic analysis [35]. Excerpts from French language interviews were translated into English after being initially coded in French. We followed three key steps in our coding and analysis strategy. First, we gained familiarity with the interviews by reading transcripts and the interviewers’ reflection notes taken immediately after each interview was completed. Second, broader codes were applied to the transcripts, with a focus on key segments to the interview germane to this analysis. This step allowed us to organise key components of the interviews into manageable sections. Third, we worked to refine, name, and explain key themes in the data to begin to make meaning and see overarching patterns within and across men’s accounts. Given the lack of qualitative scholarship in this field, our analysis is exploratory and largely descriptive, in keeping with thematic analysis.

The next stage of the analysis involved sharing the organized findings with the author team for initial input. Once this feedback was received, Grace, Gaspar, and Klassen reviewed findings using two additional rounds of iterative analysis and synthesis to further conceptualize and organise the study findings. These findings were then reviewed again by the entire authorship team for further comment to strengthen interpretation, including addressing nuances across provincial contexts.

## Results

We interviewed a total of 39 HIV-negative GBM in Vancouver (*n* = 15), Toronto (*n* = 13), and Montreal (*n* = 11). Within this sample, 64% identified as white; 21% as South Asian or East Asian, 5% as African, Caribbean, or Black, 5% as Latino, 3% as Indigenous, and 3% as Middle Eastern. 79% of the men identified as gay, 15% as queer or other, and 5% as bisexual. 87% of participants identified as cisgender men, 8% as trans men, and 5% as gender non-binary. 13% of participants were under 25 years old and 26% were over 50. Just over half of participants (56%) indicated that they had donated blood in the past when they were eligible (i.e., before they started having sex with men); for six of the participants, these experiences occurred outside of Canada.

Below, we outline a range of perspectives on plasma donation. We start with a summary of participants’ understanding of plasma donation, then progress to their general willingness to donate plasma, and conclude with an analysis of their perspectives on plasma versus blood donation policies.

### Limited understanding of plasma donation

Participants’ knowledge and understanding of plasma donation varied, but they generally expressed uncertainty about both the process of donating plasma and the utility or importance of plasma donation. While several men knew what plasma was, most articulated having only a vague understanding of it and being unfamiliar with how the process of plasma donation differs from blood donation. For example, one participant stated that, “I don’t know much about it, but I’ve heard of it” (30s, Toronto). A few participants noted they were unaware that it was possible to donate plasma separately from whole blood. Perhaps not surprisingly—and likely in keeping with the general population—participants had minimal knowledge of plasma donation with many not having given plasma donation much, if any, thought prior to the interview.

Overall, the men we interviewed were also uncertain about what plasma was used for and the demand for plasma in comparison to whole blood or what people thought of as “regular” blood donation. For example, one participant noted that, “I do not know what is precisely the use of plasma. If we take plasma, it is because there’s a good reason, a utility?” (50s, Montreal). While participants assumed that plasma was generally useful, they were unsure of how important plasma donation was from the perspective of the blood supply and blood recipients, which made some less interested in donating plasma. Indeed, one participant explained that his willingness to donate plasma was largely dependent on whether he “knew that there was a huge need for it [or not]” (30s, Vancouver). Some participants were reluctant to take a stance on plasma donation because of their limited knowledge of the process and its benefits to plasma recipients. As one man asserted, he wanted “to know more about what the nature and the need is, etcetera, and the extent of the commitment I would be making” (60s, Vancouver).

### General willingness to donate plasma: altruism vs. inconvenience

Many participants expressed a willingness to donate plasma, with one participant describing plasma donation as “fantastic” and something that he would consider doing if able (30s, Toronto). For some men, willingness to donate plasma was motivated by their perception that this was an altruistic act of civic engagement—a theme many men also expressed in relation to blood donation [6]. They viewed plasma donation as a means of contributing to the health of others in need and potentially saving lives. As one participant noted, when compared to whole blood donation: “It’s still saving a life and helping someone and whatnot so why not?” (60s, Vancouver). Some participants who said that they were willing to donate plasma argued that the altruistic benefits of plasma donation outweighed any personal time commitments associated with donation.

Conversely, several participants were less willing to donate plasma, with many emphasizing the increased time commitment and inconvenience associated with plasma donation as a major barrier. When told that plasma donation took longer than whole blood donation and that plasma donors were expected to donate regularly, many men were dissuaded from the possibility of donating plasma. One participant responded to this by noting that while donating plasma “sounds cool…in terms of especially the regular visits and the fact that it takes an hour to an hour and a half, I would rather donate blood” (20s, Vancouver).

Finally, a few men said they were uninterested in plasma donation due to the “inconvenience” or “commitment” associated with long donation visits and/or repeat visits. In reflecting on the importance of returning for future plasma donations—because plasma is held back for future confirmation of HIV status—one man expressed: “I understand why they’re doing it but I just still think it’s strange [to donate plasma again] in order to prove or in order to ensure that your blood is safer when you know you’re, say, HIV negative anyway” (Montreal, 50s). This participant echoed a recurring theme we observed in our blood donation interviews more broadly—that many men were confident in their HIV-status and critical of policies that seemingly contradicted what they personally knew about HIV testing and their own risk of exposure [5].

### Plasma donation policy views

Participants’ perspectives on a modified policy that enabled MSM to donate plasma, but continued to limit their ability to donate whole blood, varied, with some participants viewing this potential policy change in positive, progressive terms, while others viewed this policy alternative with significant skepticism. Participants’ policy perspectives were closely associated with their willingness to donate plasma.

#### Positive perspectives: plasma donation as a “stepping stone”

Many participants viewed a plasma-based policy alternative for MSM in positive terms, with some participants referring to such a policy as “a step in the right direction” (30s, Toronto), a “stepping stone,” and “a great alternative for now” (20s, Vancouver). For these men, a plasma-based policy alternative for MSM was not ideal but was an improvement that would hopefully open the door to a more inclusive MSM blood donation policy in the future. While not perfect, some participants saw this potential policy alternative as one which was less discriminatory toward GBM, by not systematically excluding them as a group, with one man stating, “what is interesting with plasma is that they are saying, yes, there is a possibility to include [gay men]. We found a way [to include you]” (50s, Montreal). For others, this policy shift did not redress feelings of exclusion but improved the likelihood that a more equitable, gender-neutral, behavior-based blood policy would be created in the future.

One participant made the argument that giving GBM the ability to donate plasma could help GBM advocate for the right to give whole blood by demonstrating that members of this group can donate safely: “I see it as a positive. It’s a start…I think that maybe, with this example, we could show that finally…it can work for blood as well” (30s, Montreal). Similarly, another man situated plasma donation as part of “incremental” progress towards a more equitable blood product donation policy:

I mean it’s okay. I mean it’s not the full opening up from the blood supply but that could be one way of moving forward and saying, ‘Okay we’ll try this for a year or two,’ and then open it up to the general public if everything stays clean and everything is good and we have no issues (40s, Vancouver).

#### Skepticism: open to plasma donation but critical of creating “second-class” donors

In contrast to the accounts of progress above, many other participants viewed a plasma-based MSM policy alternative with skepticism and from a somewhat critical perspective as they argued that it did not go far enough toward eliminating the perceived discrimination inherent in existing MSM blood donation policies and was “still not fair” (20s, Toronto). Although many of these participants reported that they were still potentially willing to donate plasma, they were more critical of a policy change in which only plasma, but not blood, would be allowed. For one participant, who was “somewhat” willing to donate plasma, this alternative was a “step in the right direction” but it still created a “two-tier system” in which GBM would only be allowed to donate plasma and not whole blood (30s, Vancouver).

Many of these participants framed blood donation as a right—that is, GBM should have the same option to donate as other potential donors—and they therefore viewed plasma donation as a positive but inadequate advance in blood product donation policy. According to these participants, granting GBM access to plasma donation, but not whole blood donation, ultimately rendered GBM in a “second-class donor” category (60s, Vancouver) subjected to different restrictions compared to the heterosexual public. One man who stated that he would consider donating plasma but that he still viewed this plasma donation option as inequitable put it like this: “Yeah, I’d prefer they had the same policy [for both blood and plasma] for both [GBM and heterosexual people], right?” (60s, Vancouver). For this participant, a fair blood product policy was one that was equally applied to all potential donors without singling out and othering GBM. In short, this participant was not satisfied with a more equitable plasma donation policy while a stigmatizing and othering blood donation policy remained—a tension we now turn to in greater detail.

#### Negative perspectives: plasma donation as “still stigmatizing”

Some participants were very critical of this policy alternative and, as such, said that they were unwilling to donate plasma should it become an option. For these participants, a plasma donation option for GBM did not resolve the fundamental underlying issues with Canada’s blood operators or with current blood donation policies, which left these GBM feeling angry and discriminated against. These men described a plasma-based policy alternative as being “still stigmatizing” (30s, Vancouver) and replicating the “us and them” (20s, Toronto) logic of past donation policies that constructed GBM as inherently risky. Another man said he was unwilling to donate plasma “out of a sense of protest” (40s, Vancouver). Speaking of these unresolved, underlying issues, one participant stated:

I think I’d probably still abstain from donating [plasma]. I think I wouldn’t necessarily feel any significant changes had been made in terms of the culture. So, while it would be something I would probably be able to do physically, it still wouldn’t have emotional or cultural safety for me to put myself in that situation, especially having to go repeatedly to demonstrate that my samples are good enough or safe enough (30s, Vancouver).

This man felt that a plasma-based policy alternative inclusive of MSM failed to adequately address the stigma and homophobia associated with Canada’s blood operators and existing MSM deferral practices, which continued to make him feel unsafe—especially as a trans man—and unwilling to donate.

Summarizing the negative attitudes of some GBM about this plasma policy option, another participant stated:

I’d still say like, okay but you’re setting an unfair precedent on gay men where you’re not doing it on straight couples. I’d still be sitting there, kind of like an asshole about it. To me, I don’t see why there has to be any difference between either demographic and so if someone asked me, “Oh, you can donate plasma,” I’d still be that dick on the street like, “How dare you? I should have full rights” (20s, Toronto).

Since this alternative plasma-related policy failed to fully reconcile the issue of equity, some participants who viewed blood donation as a right were frustrated by this insufficient potential policy change and unwilling to donate plasma. This last participant account raises a significant question about the potential unintentional consequences of a plasma policy change, including if it could potentially result in some GBM feeling worse about their inability to donate blood.

## Discussion

Our participants’ interest in plasma donation and their general acceptance of a policy where they could donate plasma but not whole blood, underscores the desire of many within the GBM community to help others. While some participants who were critical of an MSM-inclusive plasma donation option in the context of a discriminatory blood donation deferral were still willing to donate plasma, others explained that they would not consider donating plasma should they become eligible. Our data reveal a significant *plasma policy disjuncture—*a gulf between the *critical importance* of plasma donation from the perspective of Canada’s blood operators and patients and the feelings of many GBM who viewed this form of donation as somehow *lesser than or second-tier* in contrast with blood donation.

As discussed earlier, it is clear that Canada’s blood operators frame plasma donation and increasing the secure and safe supply of Canadian plasma as extremely important for the health of Canadians. Thus, from the vantage point of blood operators, a plasma donation policy that allows GBM to donate plasma, even if they cannot donate whole blood, is not considered to be a lesser contribution or second-tier form of donation. Canadian Blood Services has produced an array of resources under its *Plasma for Life* webpage to help educate potential donors about how plasma donation works, donor eligibility, commitments to patient safety, and where one can provide a non-renumerated donation—messaging that has been further updated in the context of COVID-19 and the need for convalescent plasma donors [16]. Canadian Blood Services also communicates the importance of plasma donation by highlighting patient accounts of gratitude to plasma donors, underscoring the critical and lifesaving importance of this less understood form of donation [17].

Unsurprisingly, despite these targeted education resources, most participants had limited knowledge and understanding of plasma donation and were unsure of the extent to which plasma was needed. Our findings confirm Vesnaver et al.’s hypothesis that many GBM, as well as the general population, have likely not been educated or made knowledgeable about plasma donation, including differences between the blood and plasma donation process [32]. While our finding of low knowledge is not unexpected, it is significant given the dearth of empirical scholarship regarding GBM’s views of plasma donation. The lack of understanding made some men unsure about becoming a plasma donor. Our findings are consistent with research by Bagot et al. [11] who found that for people who had never donated plasma before, being unfamiliar with the process of plasma donation and how plasma donation benefitted the blood supply were deterrents. These findings build upon our earlier work which has documented confusion among some GBM on the rationale for applying a time-based deferment for blood donation [5].

In order to successfully implement a revised policy for eligible GBM to become plasma donors, more education regarding plasma will be required; public education is a core responsibility of Canada’s blood operators [36]. Efforts at increasing plasma literacy should address how it differs from blood donation (including the level of time commitment donors are making), why plasma is medically important, and why it is possible for “low risk” MSM to donate plasma and not whole blood. Furthermore, new donation policies must be communicated in ways that do not further stigmatise and “other” GBM, or that will mark certain GBM as “safer” than other GBM. That is, the communication of eligibility under a new policy program is a highly sensitive issue that requires careful consideration and consultation with diverse GBM communities.

Our findings have implications for blood operators globally who are considering changes to plasma donation policy that would allow MSM to donate. In the Canadian context, these results are significant for Canada’s blood operators should a modified plasma donation policy allowing MSM to donate move forward. Plasma donation policy reforms being considered for MSM in Canada are occurring within the shadow of blood donation policies and a deeply fraught policy history [26, 37]. The narrative accounts of participants reveal that a plasma-specific policy change would be viewed by many GBM as insufficient to resolve fundamental feelings of unjust policy discrimination by current blood donor deferral policies that are not based on sexual behaviour. For these participants, current blood donor polices are group stigmatizing and ill-informed (by not considering risk in sexual behaviours) leading to an ‘othering’ of this population [5]. Of course, while redressing historical wrongdoings is not the stated aim of modified plasma donation policy, when thinking about themselves as potential plasma donors, GBM thought about this opportunity to donate (plasma) in relation to unjust exclusions (blood).

Participants’ narratives reveal the importance of understanding policies in relational and social terms. We argue that any plasma reform efforts for MSM that ignore the historical context of blood donation deferral, will likely fail. GBM’s accounts reveal that bracketing any policy reform activity from its political and social history undermines the goals of Canada’s blood operators from the perspective of many GBM. While many men were not satisfied with a modified plasma donation policy as an alternative to a more comprehensive reform of blood product donor policies, some viewed it as a positive, transitory, and perhaps necessary “stepping stone” toward more inclusive donation policies for GBM that are gender-neutral and based on specific behavioural practices [5]. These findings from a sample of GBM from across three Canadian cities support earlier research with GBM in Montreal who also expressed the desire for blood donation policy reform beyond plasma specific policy changes [23].

Any reforms to plasma donor screening should be done in concert with a commitment to address MSM blood donor policies and base policy reforms on the best available scientific evidence [27, 38]. Furthermore, new plasma donation processes (i.e., those that allow more MSM to donate) must be set up as to allow the collection of necessary data in order to inform subsequent changes to blood donation policies; the GBM community must also be engaged in sustained consultation on these plans and designs.

Developing the screening procedures and questions for “suitable”/“low risk” MSM plasma donors requires attention to the legacy of blood donor restrictions and considerable research and community consultation to determine how to ask questions of potential plasma donors (e.g., in relation to relationship status and number of sexual partners). Indeed, many study participants understood themselves as “low risk” potential blood donors, constructions which were largely consistent with quantitative behavioural data that were collected [6]. However, it is possible, and probably likely, that differences exist between how GBM construct their “low risk” for HIV (e.g., use of condoms, PrEP, and/or HIV “treatment as prevention”)—and why they would make sense as a safe blood or plasma donor—and the eligibility criteria that may be used to screen potential plasma donors (e.g., sexual monogamy). As with blood donation, some men with sexual practices that preclude them from plasma donation may consider being excluded as another form of discrimination, especially since many participants understood that all blood products were going to be tested and were unsure about the scientific rational for deferment.

Our results also reinforce the finding that considerable repair work and open dialogue with GBM communities is required by Canada’s blood operators [6]. It is essential that this repair work centres people who have faced multiple intersectional exclusions and systems of discrimination, especially within blood donation systems such as African, Caribbean and Black GBM in Canada [2, 39]. Numerous studies have underscored that one of the most significant motivators to donate plasma is being asked directly by blood collection agencies [10, 40]. However, this previous research was not conducted with groups of people that were deferred from blood donation. As such, increasing the pool of GBM donors is not simply about refining the way donor requests are made or improving the donation environment (e.g., the “conviviality” of staff and blood collection agency personnel has been identified as a determinant of donation) [41] but rather a more fundamental issue: (re)building institutional trust with diverse GBM who have experienced existing policies as discriminatory.

### Limitations

As the first qualitative study on the acceptability of plasma donation from the perspective of GBM in Canada’s three largest cities, our research builds upon the important qualitative research of Caruso et al. [23] in the province of Quebec. However, our analysis is subject to a number of limitations. Given the focus of our study on blood donation, we likely spoke with participants who were more motivated or had stronger opinions about the topic of blood and plasma donation. Our sample was also unique, in that just over half (56%) had at least one experience of blood donation over their lifetime. While these perspectives are not generalizable to all GBM, they do represent the opinions of an important group given that past blood donation practice is the best predictor of future blood or plasma donation in the general population [42].

In view of the connection of blood donation with notions of altruism and civic engagement [6, 9], social desirability may have shaped men’s accounts of being willing donors if eligible [43]. Our sample is also unique in that it comes from an existing cohort study of GBM, meaning that participants indicated a willingness to participate in an ongoing study of behavioural and psychosocial factors that are associated with the occurrence of HIV and other STIs.

It is important to note that the objective of our study was to understand GBM’s knowledge and perspectives on plasma and not to provide in-depth education or challenge participants’ accounts or framings of plasma donation. Future research could provide more comprehensive education on plasma donation in order to evaluate the extent to which the education of potential donors both increases willingness to donate plasma and addresses concerns about blood donation policy reform more broadly. Our research also complements ongoing stakeholder feasibility research into the multiple barriers and facilitators faced by GBM to donating plasma [32].

Our analysis focused primarily on questions of exclusion based on male same-sex sexual behaviour, as opposed to other experiences which may co-occur for participants and shape their perspectives regarding blood and plasma donation (e.g., gender-affirming surgeries, use of injection drugs, and migration history or travel) [2, 5, 39]. As noted above, since our interviews were conducted, the policy landscape for blood and plasma donation has shifted—from a 12-month deferment to 3-months. While this is a contextual limitation, it also presents a significant opportunity to use qualitative research to help inform the next phase of policy reforms and GBM community consultation, engagement, and reconciliation in other settings that currently have 1-year deferrals.

## Conclusion

The unfamiliarity of plasma donation for many people may make the communication of this donation option difficult, and its benefits less easy to understand, without significant GBM community engagement and education. These efforts of education and community repair work are necessary to help address the *plasma policy disjuncture* that our data have elucidated so that the critical importance of plasma donation is made clear for GBM. Plasma donor policies must be considered in relation to MSM blood donation policies over time to understand how donor eligibility practices are made meaningful by GBM in the context of historical disenfranchisement. However, an interest in plasma donation and being generally accepting of this policy proposal demonstrates the strong desire of many within the GBM community to help by donating blood products, and its historic links to notions of altruism and citizenship [6]. While many GBM might be willing to donate plasma if eligible, the accounts of participants reveal that some may be highly resistant and critical of these policy advances. Blood operators should expect resistance based on the perception of these efforts as not addressing the fundamental concern of GBM in this policy sphere: a discriminatory blood donor system. It is essential that blood operators clearly communicate that efforts to change the plasma donor policy to make more GBM eligible to donate is not a consolation prize for not being able to donate blood, but rather explain how it is part of a multipronged effort to change both the blood *and* plasma donor system to make them both based on best available science.

## Data Availability

The datasets generated and/or analysed during the current study are not publicly available due to privacy or ethical restrictions. Please contact the corresponding author for further information. The analysis was conducted independently from Canadian Blood Services. Findings from this study have been shared with the funding organization in order to inform their practices and potential policy reform efforts.

## Abbreviations

GBM: Gay, bisexual, queer, and other men who have sex with men
HIRI-MSM: HIV Incidence Risk Index for Men who have Sex with Men
MSM: Men who have sex with men
PrEP: Pre-exposure prophylaxis

## Footnotes

1 Canadian provinces and territories are able to decide the terms for compensating plasma donors. In Ontario, for example, Bill 21 - Safeguarding Health Care Integrity Act prohibits any form of compensation for any kind of blood donation [18]. However, in the provinces of Saskatchewan, Manitoba, and New Brunswick private clinics are able to offer compensation for plasma donation [19, 20]. Within Canada, plasma transfusion utilizes either (a) uncompensated donation collected from Canadians or (b) compensated donation from the United States that has been procured by Canadian Blood Services or Héma-Québec. Compensated plasma donation from within Canada is used in manufacturing drugs and other medical/biological therapies.

2 There are several key differences in plasma collection, testing, and processing that make plasma donation unique. With plasmapheresis, a higher volume of plasma can be collected each time and a person may donate more frequently since some blood components are returned into the donor. A proportion of recovered plasma, and most of plasmapheresis plasma, are sent to fractionation to produce important blood elements such as albumin and immunoglobulins. At present, approximately 90% of plasma collected by Canada’s blood operators is recovered plasma (through whole blood donations) with approximately 10% being plasmapheresis (source) donation. Due to insufficient plasma volume collected in Canada that is needed to produce plasma protein products, these plasma protein products are imported from foreign sources [17]. Canadian Blood Services has articulated its commitment to increasing plasma sufficiency in Canada, noting current shortfalls in the supply (e.g., what is currently collected only meets 13–14% of the need for immune globulin (Ig)—a high-demand plasma protein product [21].

3 As we have described elsewhere: “we refer to the target population of the current deferral policy as being for men who have sex with men (MSM), but reference the participants we interviewed as [gay, bisexual, queer, and other men who have sex with men] GBM to signify the diverse ways in which they identified themselves. MSM is an epidemiological category with policy relevance, but everyday people are more likely to refer to themselves and communities through common monikers or identities like gay, bisexual, and queer” [5].

4 This was determined by using the HIV Risk Index for men who have sex with men (HIRI-MSM) scores which measures the likelihood of MSM acquiring HIV based on sexual behaviours, health history, and other socio-demographic information [33]; 39% of participants were at higher risk for HIV (> 10) at the time of their baseline quantitative survey. This risk score is the threshold for recommending pre-exposure prophylaxis (PrEP) for HIV prevention [34].
